# Infant Understanding of Different Forms of Social Exclusion

**DOI:** 10.3390/brainsci9090227

**Published:** 2019-09-07

**Authors:** Claire Nicole Prendergast

**Affiliations:** Department of Psychology, University of Oslo, Forskningsveien 3A, 0323 Oslo, Norway; claire.prendergast@psykologi.uio.no

**Keywords:** social exclusion, social cognitive development, infant gaze behavior, preferential reaching paradigm, anticipatory looking paradigm

## Abstract

In a series of eye-tracking studies, we investigated preverbal infants’ understanding of social exclusion by analyzing their gaze behaviors as they were familiarized with animations depicting social acceptance and explicit or implicit social exclusion. In addition, we implemented preferential reaching and anticipatory looking paradigms to further assess understanding of outcomes. Across all experiments (*n* = 81), it was found that 7–9 month-old infants exhibited non-random visual scanning and gaze behaviors and responded systematically and above random chance in their choice of character and, to some extent, in their anticipation of the movement of a neutral character during a test trial. Together, the results suggest that not only do preverbal infants follow and understand third party social events, such as acceptance and exclusion, but that they also update their representations of particular characters as events unfold and evaluate characters on the basis of their actions, as well as the consequences of those actions.

## 1. Introduction

Social exclusion is a complex and painful experience of human existence. Owing to the threat that being excluded from social groups poses to one’s sense of belonging, which is important for maintaining health and well-being [1,2,3], and indeed must have posed to ensuring one’s survival in ancestral times [4,5,6], it is not surprising that human adults are particularly attuned and responsive to cues that signal exclusion in various social settings [2,4,7,8]. But just how adept are human infants at detecting these cues and understanding their associated outcomes? 

Humans are thought to develop attachment styles experientially, usually through a repeated pattern of interactions with their primary caregiver as infants [9]. Secure and insecure attachment styles accordingly develop as a function of two primitive and basic relational experiences: the *physical proximity* and the *responsiveness* of the caregiver during the first year of life [10,11,12] and become stable from around 10 months of age [13,14]. It is clear that in order to reach this developmental milestone, infants must be equipped with some kind of primitive capacity to monitor and track the quality of these relational experiences with their caregiver from birth. But the question of whether preverbal infants younger than 10 months are able to detect and evaluate these relational experiences outside of their caregiver dyads (i.e., as third-party observers of group-level social events) is an interesting one, and has not yet, to our knowledge, been addressed by empirical research.

Certainly, infants’ efforts for proximity maintenance and the attention-seeking behaviors they engage in with their caregivers conceptually map onto behaviors, such as mimicking and ingratiation, that crystallize later in development, but are also detected by infants as third-party observers by about 12 months of age [15]. Mimicking and ingratiation behaviors are generally employed to foster acceptance across most social domains [16], and operate in the service of avoiding *physical* and/or *psychological* aloneness, which would otherwise impinge on the maintenance of health and well-being. When circumstances challenge or prevent social acceptance and these behaviors do not work as intended, individuals are essentially experiencing some form of social exclusion. The experience of social exclusion can be explicit, direct and active or implicit, indirect and passive [17]. These two forms can further be distinguished by how they thwart the primitive relational goals of maintaining *physical* and/or *psychological* proximity to—and holding the attention and interest of—social others. Explicit and direct forms of exclusion (e.g., being told ‘*you can’t play with us*’ by the schoolyard bully) tend to actively deny the individual of *physical proximity* to relational others, and often imply no further access to group resources. Implicit and indirect forms of exclusion, on the other hand, are underpinned by passive behaviors such as being ignored or overlooked, that likely stem from the group’s lack of interest in the individual, and accordingly thwart the individual’s need to hold the attention of relational others. This often results in *psychological* and self-elected *physical* distance from the group and thus, limited or reduced access to group resources. 

With this conceptualization in mind, we began our investigation of preverbal infants’ understanding of explicit and implicit forms of exclusion by running a series of eye-tracking studies with 7–9-month-old infants in the US and Norway. Infants in this age range have reached a stage of cognitive development that enables them to pay attention to what is happening around them, and in particular to social information [18,19], but have generally had very little exposure to social events taking place outside of the family home, such as witnessing group-level social exclusion. Since our sample of 7–9 month-olds participated in the study with their parents on weekdays during working hours in the US, and parental leave lasts at least 11 months in Norway, their primary care and social environments at the point of testing had been their family homes. As such, these infants were ideal candidates for this empirical work that sought to uncover what humans may be wired to expect, as third-party observers in broader social contexts, before group-level processes of socialization have shaped any such expectations. Indeed, as infants begin to navigate their own social worlds in kindergarten and school at a later stage of development, cultural and experiential layers likely influence their evaluations of these kinds of social events. After sustained exposure to broader social contexts in childcare facilities or playgroups, toddlers may even become equipped to offer elaborate verbal explanations as to why social exclusion has occurred (e.g., ‘*John was not allowed to play with us anymore because he stole Sally’s toy*’). However, with our preverbal infant sample, we assumed that no such exposure had taken place and thus, relied on our analyses of infant gaze behavior and other infant-friendly paradigms to assess whether 7–9 month-olds could evaluate group-level social events and what preferences and expectations they may have in relation to social acceptance and exclusion events.

In this study, we created simple animations with shapes depicting third-party social acceptance and explicit or implicit social exclusion and familiarized infants with these scenes. We then used preferential reaching and anticipatory looking paradigms to assess infants’ understanding of the events they had seen and their associated outcomes. In addition, we conducted an exploratory analysis of infants’ gaze behavior and visual attention during familiarization trials and outcome scenes. We focused on the distribution of fixations on areas of interest (AOIs; the characters and the group resource shown on-screen) and compared proportions of looking time to primary characters across conditions and outcomes. Gaze transition matrices were created to explore areas of attentional synchrony (i.e., periods of time when infants’ gaze transitions were aligned) and Markov models computed to calculate the probability of transitioning fixations from one AOI onto itself or to another AOI. In addition, entropy values were calculated for these transition matrices. The computation of standardized entropy values from 0 to 1 facilitates classification of scanning behaviors [20]. Lower entropy values suggest more dependencies between the fixations, while higher entropy values suggest a more random scanning pattern [21,22]. 

Our main findings are as follows. In Experiment 1, it was found that after being familiarized with animations depicting explicit or implicit exclusion, infants responded systematically and above random chance in their choice of one character over another (i.e., the *excluded* character or the outermost group member, herein referred to as the *group gatekeeper*) during a preferential reaching task, as a function of the form of exclusion they had seen. In Experiment 2, infants were again familiarized with animations depicting acceptance and either explicit or implicit exclusion, and we used anticipatory looking in a test trial as our main outcome measure, hypothesizing the same outcomes as in Experiment 1. It was discovered that infant expectations for a neutral third-party character who had been present onstage throughout all scenes, to move toward the excluded character or the group character during the test trial, only mapped onto the same reaching preference as exhibited by infants in the implicit exclusion condition of Experiment 1. There was no clear expectation of the direction of movement of the neutral character in the explicit exclusion condition. However, it is argued that small design differences between experiments and other features of Experiment 2 may explain this outcome. Overall, the eye-tracking data collected across both experiments provided an unbiased measure of 7–9 month-old infants’ ability to make visual connections between characters as events unfolded and further increased confidence in the outcome measures used, in particular the preferential reaching task in Experiment 1. The results from Experiment 2, although not as clear, raise some interesting questions relating to the interpretation of paradigms commonly used to assess infant cognition in this way and generate a set of exciting new research questions that point to the integration of models of infants’ theory of mind—specifically in relation to the potential mismatch of infants’ expectations of the goal-directed actions of third parties and their own preferred course of action.

## 2. Experiment 1

In Experiment 1, we predicted that when given a choice between the *excluded* character and the *group gatekeeper*, more of the infants familiarized with an explicit form of social exclusion would choose the *excluded* character during a subsequent preferential reaching task. Conversely, we hypothesized that if infants were familiarized with an implicit form of exclusion, more would choose the *group gatekeeper* over the *excluded* character. We compared patterns in viewing behavior across participants and explored whether differences in gaze behavior may have led to infants’ eventual preference for the *excluded* or *group gatekeeper* agents across explicit and implicit exclusion conditions. Experiment 1 was pre-registered on the Open Science Framework (OSF) [23].

### 2.1. Method

#### 2.1.1. Participants

We tested 52 healthy infants (25 male and 27 female) ranging in age from 7 months 0 days to 9 months 28 days (*M* = 8 months 29 days, *SD* = 25 days). Parents provided informed consent for their infants to participate in the experiment. The experiment was approved by the Institutional Review Board. Infants were randomly assigned to the explicit or implicit exclusion condition. A total of 14 infants were excluded due to not meeting the strict criteria set out in the pre-registration for inclusion in the final sample [23]. These were comprised of fussiness during, or subsequently to, the familiarization trials or insufficient attention during trials resulting in failure to complete a minimum of four familiarization trials in total, and a minimum of two during which they had seen the explicit or implicit exclusion event taking place. Another 3 infants were excluded due to technical or experimenter errors. Five infants were excluded from the analysis of the preferential reaching task due to reaching for both characters at once, lunging forward and successfully managing to grab one character before evaluating both characters, or not reaching at all when prompted a maximum of three times. A sixth infant was excluded from the reaching task due to having an established color preference, as reported by the parent upon learning the color of the chosen character. This left a total of 29 infants in the reaching task analysis (15 male and 14 female), ranging in age from 7 months 0 days to 9 months 19 days (*M* = 8 months 14 days, *SD* = 25 days). However, 9 of the infants excluded from the reaching task analysis due to task error or fussiness that occurred just before or during the task provided good quality gaze data throughout familiarization trials and are thus included in the exploratory analyses of gaze behavior across conditions. Finally, gaze data from 3 infants included in the reaching task was not of sufficiently high quality for analysis (i.e., no fixation or saccadic data could be computed) and was thus excluded from the general gaze data analyses. The total sample for gaze data analysis thus consisted of 35 infants (19 male and 15 female) ranging in age from 7 months 0 days to 9 months 24 days (*M* = 8 months 24 days, *SD* = 26 days).

Infants were recruited from mailings to families in the Cambridge/Boston area whose contact details were sourced from public birth records at local town halls. The participants’ parents were contacted by phone and were given $5 compensation for travel expenses and infants received a toy to the value of $5 for participation. 

#### 2.1.2. Apparatus and Stimuli

We used a Tobii T60 eye tracker with an inbuilt monitor (17” TFT, 1280 × 1024 pixels), sampling at 60 Hz in a controlled laboratory setting. Infant eye-tracking modules were selected in Tobii Studio to track infant gaze from an approximately 50–60-cm distance from the eye-tracker. The eye tracker’s inbuilt user camera captured video footage of the infant during testing. An additional video camera recorded the infant from just left of the monitor and this footage was used to validate the reaching task that was cross-checked by two research assistants who were unaware of the theme or purpose of the study.

**Figure 1:**
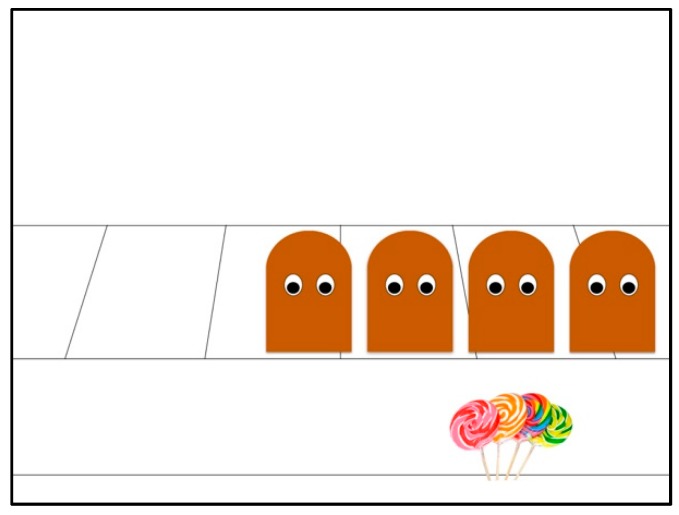
Acceptance scene outcome.

**Figure 2:**
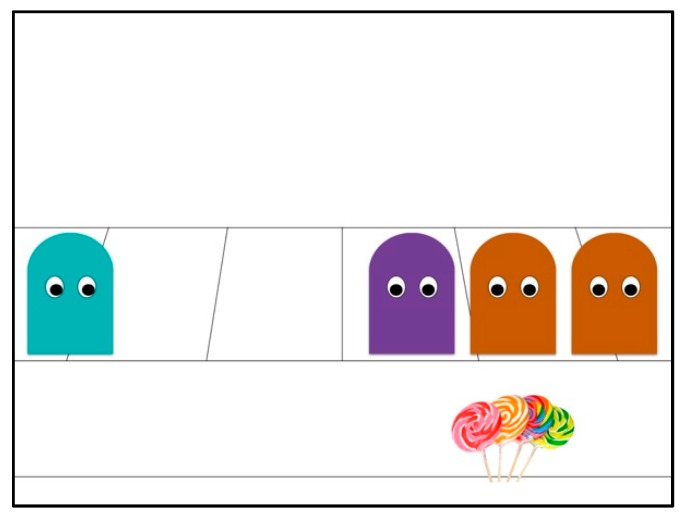
Exclusion scene outcome.

**Figure 3:**
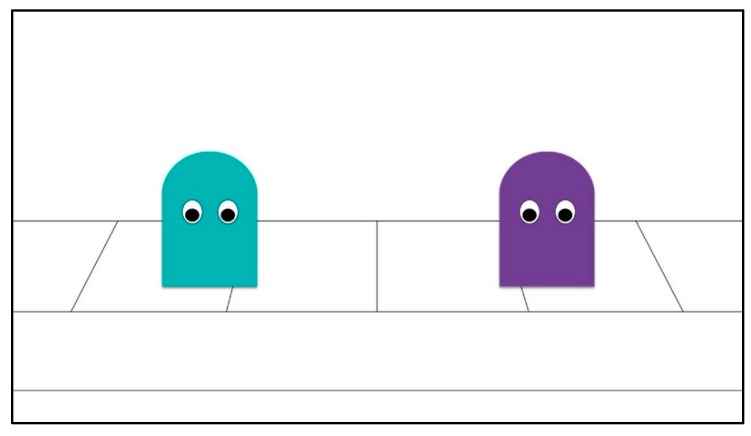
End Scene.

Animations depicting social acceptance and exclusion were created using Keynote [24], shown in Figure 1, Figure 2 and Figure 3. Three colors were selected for the animation characters on the basis of their differentiation, up to the color-blind level of tritanomaly (<0.01%). All events were spatially and temporally aligned with a small offset of 300–1000 milliseconds in the timing of events, to allow for different actions across acceptance and exclusion scenes. All the animations were exported from Keynote and converted to .avi files using MPEG Streamclip [25], trimmed at 48 s in length, and given a final resolution of 1280 × 720 px to fit to screen.

Tobii Studio 3.0.9 [26] was used to run the experiment. Raw gaze data files were exported from Tobii Studio. Recent research suggests that Tobii Studio data filters such as the I-VT filter are not suitable for lower quality data collected from infants due to head movement etc. Thus, the ‘gazepath’ package in R [27,28] was used to compute fixations and saccades in our data and the fixation durations herein are reported on that basis. However, it was noticed that infants tended to exhibit gaze aversion from the screen by turning their heads away at times, thus interrupting tracking. This was especially noticeable for those familiarized with explicit exclusion when the exclusion event was cued in the second and third familiarization trials. Therefore, gaze validity data provided by Tobii Studio formed the basis of this analysis and low gaze validity data for consecutive periods of time exceeding 500 ms constituted infants’ purposeful looks offscreen. As it is not possible to directly account for purposeful looks offscreen using the gazepath package in R, and seeing as though the accuracy of fixation data is relatively unimportant for the analysis of scanpath sequences, so long as the AOI has been visited in some way (saccadic, fixation or smooth pursuit), the analyses of scanpath sequences and areas of attentional synchrony are based on the fixation data produced by the I-VT filter in Tobii Studio. On the other hand, the transition matrices used to compute entropy values were derived using the gazepath fixation data due to the focus being on gaze transitions taking place on-screen only.

Areas of Interest (AOIs) on the stimuli were specified using Blickshift Analytics [29]—where possible, to one degree or approximately 40–50 screen pixels. All the characters and objects on-screen were defined dynamically as AOIs to allow for the spatiotemporal mapping of gaze to scene events. Blickshift Analytics was also used to correct for calibration errors that occurred during testing that had caused some data points to be slightly offset. The calibration accuracy of the eye-tracker was first checked by calibrating 5 points on the screen with an adult. Given that there were no issues with this calibration, any offset in the data was assumed to be due to errors that occurred during infant calibration. As such, the scanpaths of the infant data were superimposed on the stimuli in Blickshift Analytics, and slight adjustments were made to the gaze coordinates until they were within the most probable AOI region on the screen. Although manual, this method is particularly reliable when dynamic stimuli are used, as gaze is most likely to fall on moving objects, which helps to pinpoint and re-calibrate all data points on the stimuli more accurately. 

#### 2.1.3. Events 

The acceptance scene included three agents of equal size, color and shape (rounded archway/oval face) who formed the group. These shapes were all brown in color and had minimal features (i.e., only eyes to indicate gaze-directed action). A group resource (lollipops) was placed just below the group. An image of lollipops was used as a visually appealing object that was shared or not shared by the group as a function of acceptance or exclusion events. In this sense, it forms part of the abstract representation of characters, events and objects in the animations that have an ethological basis.

In all the scenes, the group first bounce together two times to depict group synchrony. Next, in the acceptance scene, the goal-directed action of proximity-seeking is depicted, as specified by the directional gaze and physical movement toward the group of a fourth individual brown character who enters the scene. This fourth character reaches the group and initiates a call-response communicative act [30] with the *group gatekeeper*. Social acceptance is specified as an outcome via group contingent actions and synchrony, such that all agents begin bouncing together simultaneously on-screen [31]. The group resource is also shared by the group and comes into equal access of all group members [32].

The animation depicting explicit exclusion is specified as a function of the attachment primitive of proximity, such that an act of denial of *physical proximity* to the group is perpetrated by the *group gatekeeper*. In this scene, a purple/turquoise agent plays the role of *group gatekeeper* forming the group together with two of the original group agents. Again, a fourth individual character (turquoise/purple in color) enters the scene, approaches the group and tries to initiate a call-response act as in the acceptance scene, but is instead physically pushed away from the group by the *group gatekeeper*. This results in the *excluded* character remaining stationary at a physical distance from the group, while the group bounce together in synchrony. The implicit exclusion condition, on the other hand, is represented by a passive act (i.e., a lack of *responsiveness*) where the *group gatekeeper* simply does not acknowledge the presence of the fourth individual character, nor its attempt to initiate the same call-response communicative act upon reaching the group. This results in the individual character moving away from the group by itself and remaining stationary at a physical distance, while the group bounce together in synchrony.

The colors of the *excluded* character and the *group gatekeeper* were counterbalanced between-subjects. The color of the *group gatekeeper* in the acceptance scenes remains brown so that the purple/turquoise *group gatekeeper* from the exclusion scenes does not have dual role of acceptor and excluder, that would potentially confound the choice of character during the preferential reaching task.

Finally, in both conditions, after familiarization with the acceptance and exclusion events had concluded, a final static image of the *excluded* character and the *group gatekeeper* was shown on-screen for 8 s (see Figure 3). 

Computer synthesized sounds were used throughout all scenes to accompany the movements and events taking place (e.g., a rising tone was sounded as the individual character entered the scene and again when as it approached the group).

#### 2.1.4. Procedure

Infants either sat in a secure high chair or on their parent’s lap, about 50–60 cm from the eye-tracker. Parents were asked to refrain from interacting with the infant during the experiment and to close their eyes for the duration of the experiment. A popular infant-friendly cartoon was playing on the monitor as the infants were seated, allowing time to adjust the seating parameters while infants remained distracted by the display. Once the experimenter was happy with the setup, a calibration procedure for the eye-tracker followed, whereby a black dot moved and pulsated at 5 different locations on the screen picking up the infant’s gaze at these precise points. This procedure increases the accuracy of the gaze data collected during the experiment [33]. 

After the calibration procedure was completed, infants were then familiarized with the acceptance scene followed by the explicit or implicit exclusion scene. This sequence pair repeated 2 or 3 times for a required minimum of 2 trials for each sequence pair and a maximum of 3 trials. Each trial was delimited by a black screen with a 2000-ms attention grabber before moving onto the next trial. Following familiarization with these scenes, the final static image with the *excluded* character and *group gatekeeper* was shown on-screen for 8 s, at which point a system sound signaled to the research assistant, who was blind to the condition and color of the characters, to approach and momentarily distract the infant by turning the high chair away from the screen. Meanwhile, the experimenter placed a cardboard representation of the final scene over the screen. The research assistant then promptly redirected the infant’s attention toward the screen that was now displaying the physical cardboard representation of the characters. Next, she removed the physical characters that were lightly magnetized to the cardboard screen, turned the infant in line with the additional video camera, and conducted the reaching task. Infants were first invited to visually evaluate both agents at a distance: “Look at this one and look at this one”. The characters were then pulled slightly apart laterally to encourage gaze following to a preferred character. Once infants had evaluated both characters, they were then prompted to make a choice between characters by reaching for their preferred character, “Which one do you want?”, as the characters were brought forward within their reach. This process was repeated a maximum of 3 times if the infant did not reach for a character when prompted. Some infants lunged forward reaching for one character without first evaluating both of the characters but did not succeed in actually grasping a character. In this case, the research assistant had promptly pulled the characters back out of the infant’s reach to first ensure proper evaluation of both characters by asking the infant to look at both characters one more time before a choice could be made. When this happened, the evaluation process was repeated a maximum of three times with each infant until a valid and consistent choice was made.

### 2.2. Results

#### 2.2.1. The Preferential Reaching Task

Of the 16 infants assigned to the explicit exclusion condition, 12 infants (75%) chose the excluded character. This outcome is above random chance for a binomial distribution (*p* = 0.04), as predicted [23]. While 16 infants were intended for analysis in the implicit condition, valid data was only obtained from 13 infants before the study had to be completed due to a time restriction. However, again as predicted, 10 out of 13 infants (77%) in the implicit condition chose the *group gatekeeper* (*p* = 0.048). There was no effect of color on choice within or across conditions, with 55% of infants choosing a blue character overall (*p* = 0.71).

#### 2.2.2. Exploratory Analysis of Gaze Data.

##### Scanpath Analysis and Attentional Synchrony

Infants’ gaze behavior during all the trials suggested that they were interpreting the events taking place on-screen as social scenes. Patterns of attentional synchrony included looking from the individual character, who had just entered the scene, to the group characters and back to the individual character, all in-keeping with the expectation of social goal-related actions (i.e., for the individual character to join the group) and a call-response pattern of social engagement (i.e., that the group would respond to this goal) [30]. This exact pattern in attentional synchrony occurred in 148 of a total 198 trials (74.7%). 

A second pattern in attentional synchrony that emerged in some of the gaze data from the exclusion trials across both conditions was a tendency to look from the individual character to the group resource and back to the individual character, just before or as the individual character was approaching the group (four infants, 9%), or from the group characters to the group resource to the individual character, in any order, during this timeframe (12 infants, 34%). Another two infants scanned between the group and group resource as the individual character was approaching the group. Moreover, just subsequently to the exclusion event in the exclusion trials, eight infants (23%) made a visual connection in at least one trial between the group resource and the excluded character, as well as the group characters. A further 14 infants (40%) looked from the group characters to the group resource or vice versa during this same timeframe. Overall, these visual connections suggest that many of the infants were exploring the goal-related actions of the individual character to access the group resource through the group, while some were even anticipating a response from the group in relation to their allocation of the resource.

Finally, as a tendency for infants to avert their gaze offscreen during the exclusion trials had emerged, gaze aversion as a function of ‘offscreen’ looking times greater than 500 ms was analyzed, ruling out simple tracking error where data validity was low through manual observation of the session recordings. These periods of time were coded as an AOI in our scanpath analysis and transition matrices were computed to record the rate of infants looking ‘offscreen’ across conditions. Interestingly, it was found that infants who were watching the explicit exclusion scenes averted their gaze 1.8 times more than those in the implicit condition if they had previously been fixating on the *group gatekeeper*.

##### Proportion of Fixation Durations on AOIs

The results for fixation durations reported herein pertain to the duration of time spent fixating on a particular AOI over the total time spent fixating on all AOIs within a given scene, as expressed in proportions of looking time to a particular AOI. This approach to analysis minimizes the effect of individual differences in attention and fixation behavior, as well as eye-tracking error on outcomes and thus, in our view, was the most robust way to analyze gaze behavior with infants. 

As our focus was on differentiating between viewing behavior across the conditions of explicit and implicit exclusion, the results from the exclusion trials are reported herein. In our between-subject analysis, it was found that overall, across all exclusion trials, infants in the implicit condition spent more time fixating on the *excluded* character over the *group gatekeeper*, than those in the explicit condition. Inversely, infants in the explicit condition spent a higher proportion of time fixating on the *group gatekeeper* than the *excluded* character (P_Excluded_–P_Gatekeeper_) = 0.44, 95% CI (0.18, 0.71), *p* = 0.001. This was also the case when the analysis time frame was narrowed down, from the moment just subsequent to the exclusion event up until the end of the trial, when both conditions were otherwise identical in every way (P_Excluded_–P_Gatekeeper_) = 0.15, 95% CI (0.06, 0.23), *p* < 0.001.

An analysis of gaze behaviors across exclusion trials was next conducted by grouping the infants together according to the form of exclusion they had seen and their eventual choice of character. It was found that in the implicit condition, infants who chose the *group gatekeeper* in line with the prediction on average spent a higher proportion of time fixating on the *excluded* character than those who chose the *excluded* character in the subsequent preferential reaching task (P_Excluded_–P_Gatekeeper_) = 0.16, 95% CI [0, 0.33], *p* = 0.05. However, infants who chose the *excluded* character also looked more at the *excluded* character than the *group gatekeeper*, though not to a statistically different degree on the interaction of choice and character looked at *F*(1,18) = 2.11, *p* = 0.16. The lack of statistical significance may be more a reflection of the small number of data points available for gaze data analysis in each group. Mean proportions of fixations on primary AOIs are reported in Table 1 where it can be seen that certain tendencies likely underpinned the eventual preference for one character over another, such as those in the explicit condition who chose the *group gatekeeper*, who during the exclusion trials spent a higher proportion of time fixating on the *group gatekeeper* than those who chose the *excluded* character.

A within-subject analysis of gaze behaviors throughout exclusion trials (i.e., looking at the effect of trial number on the distribution of gaze) did not yield any statistically significant results. Gaze was distributed more or less equally across trials in both conditions.

However, when the final static image of the *excluded* character and *group gatekeeper* was presented on-screen (see Figure 3), all three infants in the implicit condition who chose the *excluded* agent (against the prediction) first looked at the *excluded* agent, while only two of the 10 infants who chose the *group gatekeeper* agent (in line with prediction) first looked at the *excluded* character, when presented with this scene (*p* = 0.008). This analysis comprised of any kind of visual hit on the AOI when the image appeared (i.e., saccadic or fixation, *n* = 13). Moreover, the probability of first looking at the *group gatekeeper* was not statistically different for those in the implicit condition who chose the *group gatekeeper* and those in the explicit condition who chose the *excluded* character in line with the predictions for each condition (*p* = 0.87), such that the form of exclusion seen did not appear to have a direct role in predicting the first point of fixation for those who chose in line or against the prediction, but the eventual choice of character nevertheless differed across explicit and implicit conditions.

Furthermore, a significant difference was found when comparing infants from the explicit and implicit condition in the overall proportion of time they spent looking at either the *excluded* character or the *group gatekeeper* when presented with this final image, such that infants in the explicit condition had spent a higher proportion of time fixating on the *group gatekeeper* than those in the implicit condition and inversely, those in the implicit condition fixated for longer periods of time on the *excluded* character than those in the explicit condition (P_Excluded_–P_Gatekeeper_ = 0.14), 95% CI [0, 0.30], *p* = 0.05. When this was examined at the level of choice, it was found that the mean proportions of fixations, though not statistically different, were higher for those who chose against the prediction in both conditions than those whose choice was as predicted. This was also the case in the distribution of gaze between the *excluded* character and the *group gatekeeper*, where a higher proportion of fixations was linked to eventual choice in all the bar one group where it was roughly the same (i.e., those who chose the *excluded* character in the explicit condition). Mean proportions of fixations are reported in Table 2.

##### Transition Matrices & Entropy

Transition matrices were computed for the exclusion trials across conditions in Stata (Stata, 2018) using the gazepath filtered fixation and saccadic data. We calculated entropy values (Shiferaw et al., 2019) for each infant per trial, as well as their overall mean entropy across trials. It was found that during the first trial of the exclusion scenes, infants in the explicit exclusion condition exhibited lower entropy values than those in the implicit condition, suggesting higher dependencies between points of fixation on the screen (i.e., from one character to another) (E_Implicit_–E_Explicit_ = 0.06), 95% CI [0, 0.11], *p* = 0.03. However, by trials two and three, these differences had become negligible between conditions. There were no further significant differences between those who chose in line with or against the prediction across both conditions. Overall, the mean entropy value for all infants included in the analysis was M_Entropy_ = 0.62 (*SD* = 0.01), which suggests a non-random pattern of visual search behavior throughout the exclusion scenes.

### 2.3. Discussion

To date, research conducted with infants and toddlers has found evidence for early prosocial tendencies exhibited through infants’ own helping behaviors [34] or their preference for prosocial agents over antisocial agents [35]. The results from Experiment 1 provide further evidence for the presence of these same tendencies in infants as third-party observers during the first year of life. The *group gatekeeper* from the explicit exclusion scene displayed an overt and intentional act of denial of access to the group and group resource. This is represented by the physical repulsion of the individual character by the *group gatekeeper*, resulting in its physical distance from the group. Firstly, since sharing is a cue to prosocial behavior understood by infants [32], then not sharing is likely construed as a negative and antisocial act. Secondly, as infants are also first familiarized with scenes of acceptance, in which the individual character achieves its goal, and group synchrony and communal sharing of the group resource are depicted, the *group gatekeeper* character appears to have acted in a deviant way in the explicit exclusion scenes. It seems that infants have detected this, as evidenced by higher proportions of time spent fixating on this character over the *excluded* character throughout exclusion scenes and also during the time frame subsequent to the exclusion event. Thirdly, a higher rate of gaze aversion from this character offscreen when compared to infants in the implicit condition further suggests that this character is experienced as negative and aversive in nature. Lastly, the above random chance choice of the *excluded* character over the *group gatekeeper* in the reaching task implies that infants avoided choosing an anti-social character over another character, in line with *negativity bias* as documented in previous research [36].

On the other hand, while both conditions share the same outcome in terms of one character becoming excluded from the group with no access to the resource, the *group gatekeeper* in the implicit exclusion scenes does not act in an overtly anti-social way. Instead, it is possible that this character is regarded as negligent due to its non-responsiveness. In line with recent research investigating infants’ moral evaluations of agents’ intentional versus unintentional socially damaging actions [37], the infants in Experiment 1 may have evaluated the action or inaction of the *group gatekeeper* in the implicit scene more leniently than those who observed its explicitly acting counterpart, despite both events leading to the same outcome of exclusion for the individual character. As such, infant preference in the implicit condition defaulted to the *group gatekeeper* character during the preferential reaching task, as the benefits of the group affordances (i.e., access to the resource etc.) outweigh any potential benefits of affiliating with an *excluded* character. Furthermore, it is possible that the retreat of the *excluded* character in the implicit scenes came across as unwarranted or ambiguous to infants, and certainly, this might explain why it garnered more visual attention from the infants throughout the exclusion trials. In line with the higher proportion of fixations that were placed on the *group gatekeeper* in the explicit exclusion scenes, the increase in visual attention given to the *excluded* character in the implicit condition during the exclusion scenes also seems to be an indication of wariness toward a character who has acted differently or in a deviant way when compared to others (i.e., the characters in the acceptance scene). However, when the final scene of the *excluded* character and *group gatekeeper* was shown on-screen, the highest proportion of fixations on a character in general was linked to the final choice, apart from the those who chose the *excluded* character in the explicit condition, who fixated equally as much on both characters but still less on the *group gatekeeper* and more on the *excluded* character than those who chose the *group gatekeeper*. Interestingly, this tendency seems to suggest that some amount of focus on a particular character can perhaps indicate a wariness toward that character due to their actions, but an extended amount of time fixating on a character could consequently be indicative of added interest and thus, an eventual preference. This feature shares the same systemic properties as the theory of *differential susceptibility* [38], where genetic and phenotypic factors may be responsible for certain individuals moving beyond a specific evaluation point, and with respect to our results, in the terms of a transition from cautious evaluation of a character to preference formation for that character.

Finally, the differences in gaze behavior across conditions provide evidence for preverbal infants’ already fine-tuned ability to follow and understand third-party events relating to social acceptance and explicit and implicit forms of social exclusion. On some level, this is unsurprising given the conceptual overlay of their own primitive relational experiences with their caregivers to those of the group-level events depicted in the animations that were specified as a function of the attachment primitives of *proximity* and *responsiveness*. However, infants’ nuanced evaluation, as third-party observers, and their systematic choice of character across two distinct types of social exclusion is perhaps the most remarkable finding, given the mean age of the sample was just 8 months 14 days.

## 3. Experiment 2

In line with the predictions made for Experiment 1, we expected that most infants would anticipate that a neutral background character present onstage throughout all acceptance and exclusion scenes would move toward (‘choose to affiliate with’) the *excluded* character, if infants had been familiarized with explicit exclusion. Conversely, we hypothesized that more infants, if familiarized with scenes depicting implicit exclusion, would in turn expect the neutral character to move toward the group in a final test trial. Experiment 2 was pre-registered on OSF [23].

### 3.1. Method

#### 3.1.1. Participants

We tested 64 healthy infants in total (34 male and 30 female), ranging in age from 7 months 5 days to 9 months 29 days (*M* = 8 months 20 days, *SD* = 20 days). Parents provided informed consent for their infants to participate in the experiment. The experiment was approved by the Institutional Review Board. A total of 11 infants were excluded from the overall sample due to the strict exclusion criteria that were set out in the pre-registration on OSF [23], including fussiness or insufficient attention during familiarization trials, resulting in a failure to complete a minimum of two trials of acceptance and two of exclusion in which they had seen the specific exclusion events take place. One infant was excluded due to a technical error with the eye-tracker. Eight infants were excluded from the analysis of the outcome measure due to inattention during the critical time frame of the test trial or having no expectation of the direction of movement of the neutral character on-screen. However, 5 of these infants provided good quality gaze data across familiarization trials and are included in the analysis of gaze behaviors across conditions in general. Gaze data collected from the other 3 infants, plus an additional 2 infants, one of whom was included in the analysis of the outcome measure due to increased validity of the data at that point only, were not included in the gaze data analysis of familiarization trials due to poor and inconsistent data quality across trials (i.e., no fixation or saccadic data detected by the gazepath data filter). The outcome measure was thus evaluated with respect to data from 44 infants (26 male and 18 female), ranging in age from 7 months 5 days to 9 months 29 days (*M* = 8 months 21 days, *SD* = 22 days), while the general gaze data analysis across exclusion trials, as well as the violation of expectation (VOE), was conducted with data from 47 infants (27 male and 20 female), ranging in age from 7 months 5 days to 9 months 29 days (*M* = 8 months 21 days, *SD* = 21 days).

The first 17 infants in the sample were recruited from mailings to families in the Cambridge/Boston area as in Experiment 1, whose contact details were sourced from public birth records at local town halls. The participants’ parents were contacted by phone and email and were given $5 compensation for travel expenses. Infants received a toy with a value of $5 for participation. As a restriction on time did not allow for the completion of the study in the same geographic location, the remaining 47 infants in the sample were recruited through Facebook advertising [39] and parent-baby Facebook groups and tested in Oslo, Norway. These participants’ parents were contacted by phone and email and the infants were offered a toy or t-shirt with a value of approximately 150 NOK (~$18). The difference in the value of the reward across locations is due to different local economies.

#### 3.1.2. Apparatus and Stimuli

The first 17 infants were tested under lab conditions identical to Experiment 1. The remaining 47 infants were tested using a Tobii TX300 eye tracker with an inbuilt monitor (23” TFT, 1920 × 1080 pixels), sampled at the same frequency of 60 Hz, in an identically controlled laboratory setting. Infant eye-tracking modules were selected in Tobii Studio to track infant gaze from approximately 50–60 cm distance from the eye tracker. The eye tracker’s inbuilt user camera captured video footage of the infant during testing.

#### 3.1.3. Events

**Figure 4:**
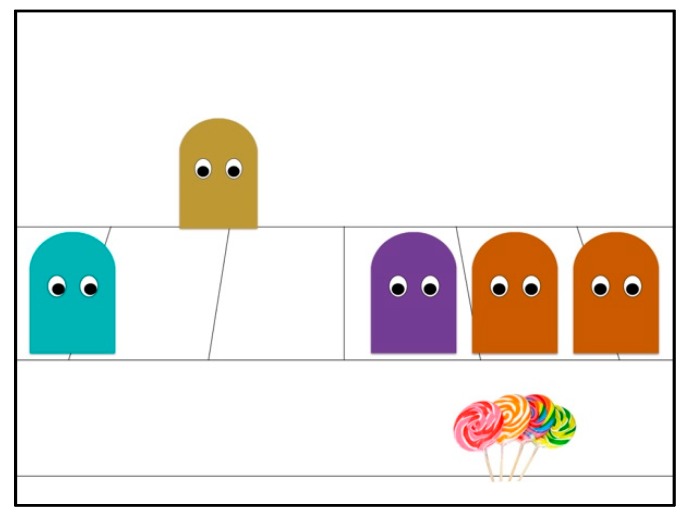
Exclusion scene outcome with neutral character.

The animations used in Experiment 2 were the same as those used in Experiment 1. Throughout all acceptance and exclusion scenes, however, a neutral beige character was present in the background of the scene, center of the excluded character’s position and the group (see Figure 4). Unlike in Experiment 1, the *group gatekeeper* remained the same color for both the acceptance and exclusion scenes, which made the connection between the scenes more salient but did, however, result in the *group gatekeeper* playing a dual role of acceptor and excluder.

After familiarization with the acceptance and explicit or implicit exclusion scenes had concluded, a final test trial was shown to capture infants’ expectations of the movement of the neutral background character on-screen (i.e., toward the excluded character or the group). To this effect, the neutral background character became activated on the stage by jiggling. It then directed its gaze to the left toward the group and to the right toward the excluded agent (order of direction randomized with eventual direction of movement) and back to center before slowly moving vertically down the stage, remaining equidistant from both the excluded character and the group. This character then stopped at a second point on the stage just above, but still in-between, the excluded character and the group. It jiggled a second time at this location and either looked toward the excluded character or toward the group (side counterbalanced with direction of movement), moving in the direction of its gaze until reaching either the excluded character or the group, upon which a bell sounded to signal the end of the scene.

#### 3.1.4. Procedure

The setup of the procedure was identical to that of Experiment 1. Infants watched the acceptance scene followed by the explicit or implicit exclusion scene in a row, 2 or 3 times for a required minimum familiarization of 4 trials and a maximum of 6. Similarly, each trial was delimited by a black screen with a 2000 ms attention grabber before moving onto the next trial. The final test trial was shown after familiarization with the acceptance and exclusion scenes had concluded. The test trial ended when the infant had purposefully looked offscreen a maximum of two times subsequent to the point in time when the neutral character had reached its final destination (i.e., the excluded character or the group) and a bell had sounded.

### 3.2. Results

#### 3.2.1. Anticipatory Looking Paradigm

Forty-four infants were included in the final analysis of the anticipatory looking paradigm. Twenty-two of these infants had been assigned to the explicit exclusion condition. Contrary to what we had predicted [23], 12 infants (54.5%) first looked from the neutral character toward the group, as the neutral character began to move vertically down the stage. The remaining 10 infants first looked from the neutral character toward the excluded character (45.5%) during this time. This outcome is not statistically different for a binomial distribution (*p* = 0.415). However, as predicted, of the 22 infants who were assigned to the implicit exclusion condition, 16 (72.7%) looked from the neutral character toward the group, as the neutral character began to move down the stage, while only 6 infants (27.2%) looked from the neutral character toward the excluded character (*p* = 0.05). There was no effect of color on direction of gaze, with 54% of infants overall first looking from the neutral character to a purple character (*p* = 0.65).

Furthermore, it was observed that some infants looked in both the direction of the group and the excluded agent during the critical time period (i.e., as the neutral agent was slowly moving down the stage). As such, Markov models were computed to calculate the likelihood of infants looking from the neutral character to the excluded character or to the group during this time frame. From the Markov models computed, the data overall supported the expectation of the neutral character’s movement, such that the overall probability of observed transitions from the neutral character to the excluded character or to the group during the critical time frame was highest for the side first looked to by the infant. One exception was the group of infants who first looked from the neutral character to the group in the explicit condition, against the prediction, who were no more likely to transition their gaze from the neutral character to the excluded character or to the group during the critical time frame (*p* = 0.13). The mean probabilities of gaze transitions from the neutral character are reported in Table 3.

#### 3.2.2. Violation of Expectation

Together with the directional anticipation of the neutral character’s movement, we aimed to test for longer looking times as a result of a violation of expectation when the neutral character moved in an unexpected direction, depending on the form of exclusion witnessed (i.e., either towards the excluded character or the group). Infants were randomly assigned to either outcome across both conditions. Data from the 44 infants included in the analysis of the outcome measure was used to analyze looking times during this time frame.

Given the slightly aversive nature of the exclusionary events previously witnessed by the infants (especially in the explicit exclusion condition) and as noted in the pre-registered report, we allowed infants to look offscreen when or shortly after the neutral agent had reached its final destination and back on-screen one more time, to allow for anticipation of a negative event resulting in gaze aversion. The time spent looking offscreen was subtracted and the log value of the total remaining looking times after 24,000 ms of the scene was compared (i.e., the point at which the neutral character had arrived at its final destination and the bell had sounded).

Of the 23 infants assigned to the explicit condition, 10 observed a final test trial in which the neutral character moved toward the group, while 13 saw the neutral character approach the excluded character. Of the 23 infants familiarized with implicit exclusion, 13 saw the neutral agent move toward the group, while 10 saw the neutral agent approach the excluded agent.

The differences in looking times overall, though not statistically significant, aligned with our expectations in the explicit condition, whereby infants tended to look longer if the neutral agent had approached the group. In the implicit condition, however, differences in log looking times were negligible with infants looking slightly longer when the neutral character approached the group. Looking times were thus longest when the neutral character approached the group in both conditions, but only to a significant degree in the explicit condition. The mean looking times are presented in Table 4 in milliseconds, together with the number of observations per group.

Where infants were looking during this time frame, depending on the direction that the neutral character had taken, together with the type of exclusion that infants had seen, as well as their expectations of the direction of the neutral character, was analyzed. Proportions of time spent fixating on particular characters from the gazepath fixation data available during this time frame (*n* = 34) are shown in Table 5, where two interesting patterns can be observed.

**Figure 5:**
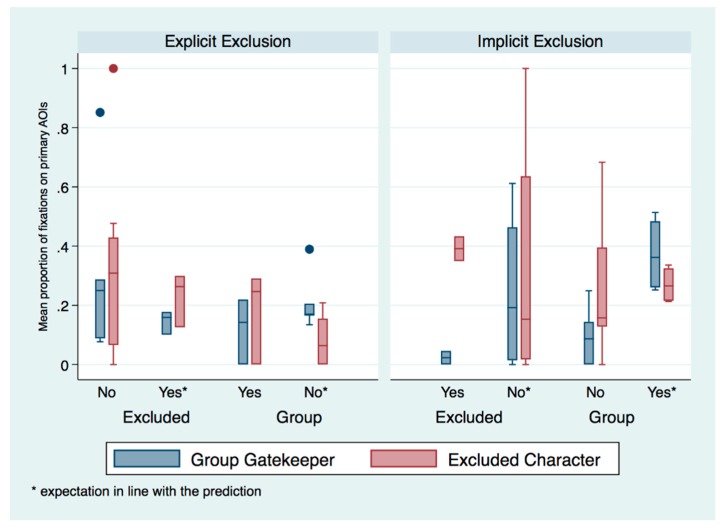
Mean proportion of fixations on primary AOIs as a function of condition, direction and expectation of the movement of the neutral character.

Firstly, in the explicit exclusion condition, those who expected the neutral character to move toward the group against the prediction, and who saw this happen, fixated less on the *group gatekeeper* than those who expected the neutral character to move toward the *excluded* character, the former group exhibiting more variation than the latter (as shown in Figure 5). Inversely, if the neutral character moved toward the *excluded* agent, those expecting this outcome fixated less on the *group gatekeeper* than those who did not expect it, the latter group exhibiting more variation than the former. This first pattern suggests an effect of the direction of movement of the neutral character and expectation of this direction on gaze behavior for those familiarized with explicit exclusion.

Secondly, infants in the implicit and explicit condition, who were anticipating the neutral agent to approach the *excluded* agent, but who instead saw the neutral agent approach the group, spent an inverse proportion of time fixating on either character, with those in the explicit condition focusing more on the *group gatekeeper* than the *excluded* agent, and those in the implicit condition focusing more on the *excluded* agent than the *group gatekeeper*. Infants in the implicit condition who expected the neutral character to move toward the excluded character exhibited greater variation in this. This second feature of the data suggests an effect of form of exclusion on gaze behavior related to the direction of movement of the neutral character. Overall, the greater variation in the proportions of fixations on different characters of those whose expectations were against the predictions we made, may suggest that their expectations, as a group, were not as uniform as those who were anticipating the neutral character to move in the direction that we predicted they would, depending on form of exclusion that had been shown.

#### 3.2.3. Exploratory Analysis of Gaze Data

##### Scanpath and Attentional Synchrony

As in Experiment 1, many of the same patterns in attentional synchrony emerged in the data. Seven infants (15%) looked from the individual agent to the group resource (lollipops), as the individual agent was approaching the group in at least one of the exclusion trials. A further eight infants (17%) transitioned their gaze to and from group characters and the group resource, and the individual agent, as the individual agent was approaching the group. Again, this implies that some infants were inferring that the goal of the individual character was to gain access to the group resource by approaching the group, while others were seemingly pre-empting how the group may respond to this goal through their subsequent visual investigation of these characters. Furthermore, during the time frame subsequent to the exclusion event in the exclusion trials, four of the same infants who had transitioned their gaze from the individual agent to the resource and group characters as the individual agent approached the group, again looked from the now excluded individual agent to the resource and back, or onto the group characters. A further two infants who had looked from the group agents to the resource and back, also exhibited this pattern of looking behavior in the final moments of the exclusion scene. An additional 18 infants (38%) looked from group characters to the resource and back in one or more of the exclusion trials after the exclusion had taken place. There was no main effect of form of exclusion witnessed on this behavior with roughly the same number of infants in both conditions transitioning their gaze in this way. However, six infants in the implicit condition who exhibited this behavior did so across two or more trials (50%), while all 11 infants (100%) in the explicit condition who exhibited this behavior only did so during one trial. One possible explanation may be that the actions of the characters in the explicit exclusion scene drew attention away from other features such as the group resource.

Finally, the same pattern of gaze aversion away from the *group gatekeeper* exhibited by infants in the explicit condition was not found, who unlike in Experiment 1, tended to avert gaze from this character offscreen a roughly equal amount of times as those in the implicit condition. One explanation may be the use of two different models of the Tobii eye trackers, both in terms of the variation in how low validity data is computed by each model (i.e., the periods of time that were marked as purposeful looks offscreen) and the difference in screen size. The screen used to test infants in Oslo, Norway was 17 cm wider than the screen using in Cambridge, MA, and although the stimuli shown on-screen was the same size, infants who aimed to avert their gaze completely offscreen may have actually landed on a peripheral area of the screen without losing the tracking before returning their gaze to the stimuli. As such, one recommendation would be that future studies looking to investigate this effect use the same eye tracker to do so.

##### Proportion of Fixation Durations on AOIs

Once more, a comparison of the proportion of fixations on particular characters and the distribution of attention across exclusion trials was completed. As in Experiment 1, it was found that infants spent a higher proportion of time fixating on the *excluded* character if familiarized with implicit exclusion and a lower proportion of time fixating on the *group gatekeeper.* Inversely, those in the explicit condition paid more attention to the *group gatekeeper* and less attention to the *excluded* character (P_Excluded_–P_Gatekeeper_) = 0.16, 95% CI (0.06, 0.25), *p* = 0.001. Unlike in Experiment 1, this difference did not maintain statistical significance for the separate analysis of the final time frame in the exclusion scenes, subsequent to the exclusion event up until the end of the trial, (P_Excluded_–P_Gatekeeper_) = 0.11, 95% CI (−0.05, 0.27), *p* = 0.17.

When gaze behavior was examined throughout exclusion trials, in terms of the expectations that infants had for the movement of the neutral character during the test trial, there were no statistically significant differences between groups in the proportions of time spent fixating on particular characters over others. Again, a lack of statistical significance in these comparisons may be more a reflection of the small number of data points available for gaze data analysis to compare each group. The mean proportions of fixations are reported in Table 6 where it can be seen that certain tendencies likely underpinned the eventual expectations shown for the direction of movement of the neutral character, such as those in the explicit condition who looked from neutral character to the group, who on average spent a higher proportion of time fixating on the *group gatekeeper* character throughout exclusion trials, much like their counterparts in Experiment 1 who chose the *group gatekeeper* in the reaching task. Similarly, those in the implicit condition who looked from the neutral character toward the *excluded* character, against the prediction, had spent more time fixating on the *excluded* character than the *group gatekeeper* across exclusion trials.

To explore these tendencies further, a comparison of the proportion of fixations that infants allocated to characters in the final test trial of Experiment 2 was undertaken. Here, the same pattern was upheld with statistical significance in the implicit condition, such that infants who expected the neutral character to move toward the group as predicted, paid more attention to the *group gatekeeper* during the test trial (P_Group_–P_Excluded_) = 0.21, 95% CI (0.01, 0.40), *p* = 0.03. While those who expected the neutral character to move toward the excluded character had paid more attention to the excluded character (P_Excluded_–P_Group_) = 0.14, 95% CI (0.06, 0.21), *p* < 0.001.

Finally, a within-subject analysis across exclusion trials was conducted, comparing results between explicit and implicit exclusion conditions to test whether, for example, infants spent a higher or lower proportion of time fixating on particular characters from one trial to the next. Again, attention was more or less evenly distributed across trials. One difference that did emerge in the data was a significant drop-off in attention toward the neutral character in trial two of the explicit exclusion scenes (*M* = 0.085, *SD* = 0.0111), while increased attention had been given to this character during the second exclusion trial in the implicit condition (*M* = 0.158, *SD* = 0.020). Thus, there was a significant main effect of exclusion condition on attention toward the neutral character during the exclusion trials, *F*(1, 88) = 3.62, *p* = 0.01, and a significant interaction between exclusion condition and trial, *F*(1, 88) = 4.78, *p* = 0.03. Again, in comparing the two exclusion conditions, this suggests that the exclusionary events shown in the explicit condition may have drawn attention away from other features, such as the background neutral character in the explicit condition, but this was not the case for the implicit condition.

##### Transition Matrices & Entropy

An analysis of entropy values computed from the transition matrices for Experiment 2 yielded no significant differences across exclusion conditions or trials. Overall, mean entropy was slightly higher than in Experiment 1 (*M* = 0.744, *SD* = 0.014). This may be more a reflection of an extra AOI being added to the matrix due to the neutral character being present on-screen, that likely led to a greater distribution of fixation points across all characters and thus, less direct connections between the main characters, as in Experiment 1.

The overall mean entropy value for infants’ gaze transitions during the test trial was much lower (*M* = 0.252, *SD* = 0.029), and while the group resource was not shown on-screen during this trial leaving one less AOI on-screen, the mean value nevertheless suggests higher dependencies between fixation points and, thus, implies a focal fixation behavior in infants during the test trial. There were no significant differences across exclusion conditions, outcomes or expectations of outcome on entropy values from the test trial.

### 3.3. Discussion

While the results of the anticipatory looking paradigm used in Experiment 2 only matched the results from Experiment 1 in the implicit condition, the lack of replication of outcomes in the explicit condition across paradigms generates some interesting questions and considerations.

One explanation for why the same result did not occur in the explicit condition could be linked to more low-level features of the study, such as small design differences. Notably, in Experiment 2, the *group gatekeeper* both accepted and excluded characters across acceptance and exclusion scenes, giving this character a dual role of acceptor and excluder. The goal of designing the scenes in this way had been to make the connection between the acceptance and exclusion scenes more salient. This was not the case in Experiment 1, where the color of the *group gatekeeper* returned to brown in the acceptance scenes and although this reduced the possible confound in infants’ choice of character during the reaching task in Experiment 1, it seemed less natural to disconnect the acceptance and exclusion scenes from one another in this way. As such, infants in Experiment 2 may have felt less wary of the *group gatekeeper* or evaluated this character more leniently due to its exhibition of both prosocial and antisocial actions, and thus that the neutral character could be expected to ‘take its chances’ and approach the group either way. This might explain why a higher proportion of fixations on this character were not present in the final time frame of the exclusion trials, for example, as was the case in Experiment 1.

A second possible confound is related to the anticipatory looking paradigm used to assess infants’ expectations of the movement of the neutral character. Across all the trials, the infants had only been familiarized with scenes in which every character who became active or appeared on-screen, approached the group. This may have led infants to expect that the neutral character, once activated would, by default, behave in the same way as every other character. While this would confound the result from the implicit condition if analyzed on its own, the lack of a significant difference in expectation of movement for infants in the explicit condition suggests that, by contrast, the explicit exclusionary events witnessed by infants in this condition pushed back on the potential low-level expectation that the neutral character would approach the group purely because all other characters had.

Certainly, given that our method was to record the first transition of gaze from the neutral character to either side, but the likelihood of looking from the neutral character toward the group was no higher than that of looking toward the excluded character during the critical time frame, if the group were first looked at, suggests that at least some of those who looked toward the group in the explicit condition may have done so because of this low-level expectation. A greater variation in the looking behavior of the two groups of infants across conditions whose expectations did not match our predictions also implies that these infants were less uniform in their gaze behavior as a group in terms of how they had interpreted the scenes and the goal of the neutral character. While the resource had been removed from the test trial so as not to confound the expectation of movement of the neutral character toward the group for this reason, one way to better align results from Experiment 1 and Experiment 2 would be to also remove the other group characters from the scene, such that the group itself would not be a confound for the default goal of approaching that same group, as witnessed during all other scenes.

Finally, the results from the VOE analysis of longer looking times, recorded from the point in time when the neutral character had reached its final destination, showed that infants looked longest in the explicit condition when the neutral character approached the group, compared to when it approached the excluded character. This aligned with our prediction based on there being a violation of expectation due to this outcome. However, the absence of a significant difference in the length of looking times in the implicit condition across the two outcomes suggests that infants may have looked longer when the neutral agent approached the group than when it approached the excluded agent, in anticipation of subsequent action from the *group gatekeeper*, again based on all previously witnessed events across familiarization trials, in which this character had responded in some way to an agent approaching the group.

## 4. General Discussion

We made our predictions for Experiment 2 on the same basis as those made for Experiment 1, in that we predicted infants would expect the neutral agent to approach the *excluded* character if familiarized with explicit exclusion, due to the perceived antisocial actions of the *group gatekeeper*, and that conversely, in the implicit condition, the neutral character would default to approaching the *group* due to the benefits of affiliating with the group outweighing those of affiliating with an *excluded* character. One key difference across the paradigms used is that in Experiment 1, infants act as agents who may choose to affiliate with either the *excluded* character or the *group gatekeeper* through their own reaching preference for one of the characters in its physical form. In Experiment 2, however, infants are predicting the actions of a third-party neutral background character. With an improved design and the removal of possible confounds for Experiment 2, it would be interesting to see if results from the anticipatory looking paradigm would align more clearly with the reaching task paradigm in both conditions. A lack of alignment in results, otherwise, could point to evidence for the role of theory-of-mind in infant preferences and expectations, such that infants may themselves choose to affiliate with a particular character, but wouldn’t necessarily assume that other third-party characters would act in that same way.

Regarding the exploratory analysis of the gaze data, while small sample sizes and noise occurring in the data (i.e., head movement, level of attention etc.) due to factors that are difficult to control for when conducting eye-tracking research with infants, make statistical comparisons between conditions non-meaningful at times, the patterns and mean differences that emerged in the data generally worked in support of the main hypotheses, as well as in support of the reaching task outcome of Experiment 1, in terms of infants’ eventual choice of character—both increasing confidence in the reaching task method and signaling an absence of transfer deficit from the screen representation of the characters to their physical form. In Experiment 2, subtle differences in gaze behavior also helped to expose some possible designs flaws in the stimuli that can now be improved upon for next stages of this research program.

Another interesting finding was that infants on average, across both experiments, spent more time looking at characters who acted differently to others or in a deviant way during exclusion trials, depending on the form of the exclusion they were familiarized with, suggesting a kind of wariness toward the character. At some point though, this visual interest, if extended, especially during the final static scene of Experiment 1 and the test trial of Experiment 2, was predictive of an eventual preference for this character or expectation that a neutral character would move toward and affiliate with this character. Studies that could replicate this effect, in general, perhaps with some investigation of the shared systemic features with *differential susceptibility*, could potentially discover a threshold that would enable the disambiguation of increased attention toward a character that is due to wariness or caution and otherwise, indicative of preference formation.

## 5. Conclusions

Across both experiments, scanpath sequences and areas of attentional synchrony found in the gaze data provide strong evidence for preverbal infants’ ability to follow the events shown onscreen and infer the socially goal-directed actions of the characters. Furthermore, their systematic evaluations of the characters and events point to an aversion of perceived anti-social characters (i.e., based on *negativity bias*) and otherwise to a preference that defaults to the group character in the absence of an explicit anti-social act. Future studies could investigate the same themes of acceptance and exclusion with older infants, both longitudinally and cross-culturally, to uncover how different processes of socialization may shape and change infant’s evaluations of these events across development.

The novel application of analytical methods used in this study, such as the calculation of entropy values for gaze transition matrices, also provide further evidence of infants’ ability to comprehend third-party acceptance and exclusion events and update their representations of the characters accordingly. In the valid gaze data collected from all exclusion trials during both experiments (*n* = 81), it was thus found that infants exhibited non-random visual scanning and gaze behaviors.

Finally, the results of both outcome measures and the factors predicting these outcomes, in terms of gaze behavior, illustrate that preverbal infants, as third-party observers, are equipped to detect social actions that cue explicit and implicit exclusion on a group level within the first year of life.

## Figures and Tables

**Table 1:** Mean proportions of fixations on primary areas of interest (AOIs) during exclusion trials.

Character	Condition	*n*	*M*	*SD*	(95% Confidence Interval)
Excluded Character	Explicit	4	0.321	0.022	(0.274, 0.368)
	Explicit *	12	0.338	0.019	(0.297, 0.379)
	Implicit	3	0.271	0.048	(0.164, 0.376)
	Implicit *	10	0.439	0.042	(0.345, 0.532)
Group Gatekeeper	Explicit	4	0.445	0.047	(0.345, 0.545)
	Explicit *	12	0.351	0.032	(0.281, 0.419)
	Implicit	3	0.230	0.058	(0.101, 0.358)
	Implicit *	10	0.268	0.022	(0.218, 0.317)

* Choice as predicted.

**Table 2:** Mean proportions of fixations on primary AOI during the end scene.

Character	Condition	*n*	*M*	*SD*	(95% Confidence Interval)
Excluded Character	Explicit	4	0.399	0.143	(0.104, 0.693)
	Explicit *	12	0.493	0.033	(0.425, 0.561)
	Implicit	3	0.714	0.117	(0.473, 0.955)
	Implicit *	10	0.433	0.092	(0.242, 0.622)
Group Gatekeeper	Explicit	4	0.601	0.143	(0.306, 0.895)
	Explicit *	12	0.507	0.033	(0.438, 0.574)
	Implicit	3	0.286	0.117	(0.044, 0.526)
	Implicit *	10	0.567	0.092	(0.377, 0.757)

* Choice as predicted.

**Table 3:** Mean probabilities of looking from the neutral character to the excluded character or group.

Direction First Looked	Condition	*n*	*M*	*SD*	(95% Confidence Interval)	*p*
Excluded	Explicit	12	0.056	0.127	(−0.025, 0.136)	0.002 **
	Explicit *	10	0.332	0.238	(0.162, 0.502)	
Group	Explicit *	10	0.414	0.301	(0.223, 0.606)	0.130
	Explicit	12	0.244	0.190	(0.108, 0.380)	
Excluded	Implicit *	16	0.351	0.198	(0.143, 0.559)	0.001 **
	Implicit	6	0.035	0.057	(0.005, 0.066)	
Group	Implicit	6	0.071	0.079	(−0.012, 0.154)	0.020 **
	Implicit *	16	0.404	0.328	(0.230, 0.579)	

* Expectation of direction as predicted. ** Significant at 0.05 level.

**Table 4:** Mean looking times at the end of the test trial in milliseconds.

Direction of Neutral Character	Condition	*n*	*M*	*SD*	(95% Confidence Interval)
Excluded	Explicit	8	11141.13	1631.58	(785.74, 14431.51)
	Explicit *	4	6983.50	2272.48	(2400.61, 11566.39)
	Implicit	2	13494.00	5102.50	(3203.83, 23784.17)
	Implicit *	7	7838.86	1316.62	(5183.65, 10494.07)
Group	Explicit	4	12538.38	2553.65	(7388.45, 17688.30)
	Explicit *	6	12547.42	2727.30	(7047.30, 18047.53)
	Implicit	4	8463.25	3357.79	(1691.62, 15234.88)
	Implicit *	9	9167.94	2483.15	(4160.21, 14175.68)

* Expectation of direction as predicted.

**Table 5:** Proportions of fixations on primary AOIs at the end of the test trial.

Character	Direction of Neutral Character	Condition	*n*	*M*	*SD*	(95% Confidence Interval)
Group Gatekeeper	Excluded	Explicit	8	0.230	0.071	(0.084, 0.375)
		Implicit *	4	0.391	0.042	(0.306, 0.477)
		Explicit *	3	0.230	0.053	(0.122, 0.337)
		Implicit	2	0.327	0.232	(0.148, 0.801)
	Group	Explicit *	5	0.179	0.090	(0.005, 0.364)
		Implicit	5	0.273	0.121	(0.027, 0.519)
		Explicit	3	0.107	0.047	(0.012, 0.202)
		Implicit *	4	0.270	0.032	(0.205, 0.335)
Excluded	Excluded	Explicit	8	0.277	0.102	(0.069, 0.485)
		Implicit *	4	0.023	0.023	(0.024, 0.070)
		Explicit *	3	0.146	0.023	(0.099, 0.193)
		Implicit	2	0.212	0.098	(0.012, 0.412)
	Group	Explicit *	5	0.121	0.064	(0.011, 0.252)
		Implicit	5	0.096	0.047	(0.000, 0.192)
		Explicit	3	0.215	0.059	(0.095, 0.335)
		Implicit *	4	0.372	0.066	(0.238, 0.506)

* Expectation of direction as predicted.

**Table 6:** Mean proportions of fixations on primary AOIs during exclusion trials by expectation.

Character	Condition	*n*	*M*	*SD*	(95% Confidence Interval)
Excluded Character	Explicit	12	0.336	0.017	(0.301, 0.371)
	Explicit *	9	0.311	0.027	(0.257, 0.366)
	Implicit	6	0.441	0.010	(0.422, 0.460)
	Implicit *	16	0.386	0.021	(0.343, 0.428)
Group Gatekeeper	Explicit	12	0.376	0.039	(0.296, 0.456)
	Explicit *	9	0.310	0.026	(0.257, 0.362)
	Implicit	6	0.219	0.037	(0.145, 0.293)
	Implicit *	16	0.253	0.038	(0.176, 0.331)

* Expectation of direction as predicted.
